# Neural Processing of Familiar and Unfamiliar Children’s Faces: Effects of Experienced Love Withdrawal, but No Effects of Neutral and Threatening Priming

**DOI:** 10.3389/fnhum.2016.00231

**Published:** 2016-05-26

**Authors:** Esther Heckendorf, Renske Huffmeijer, Marian J. Bakermans-Kranenburg, Marinus H. van IJzendoorn

**Affiliations:** ^1^Centre for Child and Family Studies, Leiden UniversityLeiden, Netherlands; ^2^Leiden Institute for Brain and Cognition (LIBC), Leiden UniversityLeiden, Netherlands

**Keywords:** threat, priming, face processing, superior temporal gyrus, inferior frontal gyrus, love withdrawal

## Abstract

In the face of a potential threat to his or her child, a parent’s caregiving system becomes activated, motivating the parent to protect and care for the child. However, the neural correlates of these responses are not yet well understood. The current study was a pilot study to investigate the processing of subliminally presented threatening primes and their effects on neural responses to familiar and unfamiliar children’s faces. In addition, we studied potential moderating effects of empathy and childhood experiences of love-withdrawal. A total of 45 students participated in an fMRI experiment in which they were shown pictures of familiar children (pictures morphed to resemble the participant like an own child would) and unfamiliar children preceded by neutral and threatening primes. Participants completed a modified version of the Children’s Report of Parental Behavior Inventory to measure parental love withdrawal, and the Empathic Concern scale of the Interpersonal Reactivity Index to measure affective empathy. Contrary to our expectations, we did not find evidence for subliminal priming effects. However, we did find enhanced activity in the right inferior frontal gyrus (IFG; involved in self-referential processing) and in face processing areas (infero-lateral occipital cortex and fusiform areas) in response to the familiar child, indicating preferential processing of these faces. Effects of familiarity in face processing areas were larger for participants reporting more love withdrawal, suggesting enhanced attention to and processing of these highly attachment relevant stimuli. Unfamiliar faces elicited enhanced activity in bilateral superior temporal gyrus (STG) and other regions associated with theory of mind (ToM), which may indicate more effortful ToM processing of these faces. We discuss the potential difference between a familiarity and a caregiving effect triggered by the morphed faces, and emphasize the need for replication in parents with pictures of their “real” own child.

## Introduction

In the face of a potential threat or danger in the environment, a parent’s caregiving system may become activated when his or her child or a stimulus reminiscent of that child (such as crying or a picture of the child’s face) is present and the threat is not overwhelmingly strong (Mikulincer et al., 2005; George and Solomon, 2008; Swain et al., 2014). Even when a parent is not consciously aware of a threatening stimulus in the environment, he or she might still process this threatening stimulus to some extent, which could lead to specific parental behaviors (with accompanying changes in brain activity) to protect and care for the child (Bowlby, 1988; Bakermans-Kranenburg and van IJzendoorn, in preparation). It has been argued that the caregiving system is complementary to the attachment system (George and Solomon, 2008; Strathearn et al., 2009), and is not restricted to the parent-child relationship but rather extends to other intimate relationships such as the relationships with siblings or partners (e.g., Mikulincer et al., 2005). In the current study we focus on the neural processing of familiar and unfamiliar faces after subliminal neutral or threatening primes. The familiar faces were created by morphing a child’s face with the participant’s own face to suggest familiarity and potentially biological relatedness in order to trigger the caregiving system.

Individuals may be able to process affective information, especially potentially threatening stimuli, fast and automatically, and possibly even without conscious awareness (Whalen et al., 1998; Globisch et al., 1999; Mikulincer et al., 2005). Since it may take hundreds of milliseconds to consciously perceive a potential threat (Koch and Tsuchiya, 2007), a system in the human brain that can react to potential threats before conscious awareness seems advantageous from an evolutionary perspective, as it enables a fast reaction that can preserve oneself or one’s offspring from danger or death. Subliminal primes can be used to examine the preconscious processing of threat-related information. In some previous studies, researchers found evidence for the human brain’s capacity to process threat-related visual stimuli without conscious awareness. For example, in one study participants rated neutral stimuli (the target) more positively when these stimuli were preceded by a subliminal prime depicting a happy face and more negatively when targets were preceded by a prime depicting an angry face (Almeida et al., 2013). Brain imaging studies also found some evidence for the brain’s ability to process threatening stimuli without conscious awareness. In these studies, researchers mainly focused on amygdala activity in response to subliminally presented angry or fearful faces. The amygdala is a subcortical structure commonly associated with the processing of emotional, especially threat-related, content (LeDoux, 1998). Briefly presented fearful (Whalen et al., 1998) and angry (Morris et al., 1998) faces evoked right amygdala activity.

However, in some studies no evidence for the existence of such an automatic processing system of threat-related stimuli was found. For example, in earlier studies with threat-related stimuli presented in supraliminal and subliminal conditions, enhanced amygdala activity was found in the supraliminal, but not in the subliminal condition (Pessoa et al., 2006; Hoffmann et al., 2012). Importantly, not everyone may respond to emotional or threatening information in the same way, and such moderating effects may explain inconsistent findings for main effects of threat-related stimuli. Considering parental responses or responses to biologically related or otherwise familiar others in threatening contexts, factors such as empathy and individuals’ own childhood experiences with their attachment figures may influence how they react to a potential threat to offspring or other familiar persons.

With regard to empathy, which has been defined as the capacity to experience and understand the emotional states of others (Eres et al., 2015), cognitive (understand), affective (experience) and imitative (action) components can be distinguished (Klimecki and Singer, 2013). In the current study, we are mainly interested in the affective component of empathy, which refers to how we feel when we imagine the emotions of another person in a particular situation (i.e., when we “put ourselves in the other person’s shoes”). This affective component refers to a mature affective response that is experienced with a certain distance to the person empathized with rather than the more primitive and potentially dysfunctional copying of the target’s affective response or distress (Davis, 1983; De Corte et al., 2007). In previous research, viewing a beloved person in pain elicited activity in brain areas associated with affective dimensions of pain (e.g., dorsal anterior cingulate cortex, dACC, see Lieberman and Eisenberger, 2015), with stronger effects in participants with high scores on empathic concern (Singer et al., 2004). In addition, observing someone experiencing “social pain” (i.e., being socially excluded) elicited brain activity in similar areas (e.g., anterior insula, anterior cingulate cortex) in highly empathic but not in less empathic participants (Masten et al., 2011). Because pain, whether social or physical, results from a harmful stimulus in the environment, we may, extrapolating from these results, expect that highly empathic individuals will react stronger to a potential threat to their child or a familiar other. It should be noted, however, that the intensity of the threat could modulate responses of caregiving and protection, since overwhelmingly strong threats might turn the focus away from the other—even when it is offspring—to protecting oneself (Mikulincer et al., 2005). However, the stimuli used in the current study depict moderate rather than extreme threats.

Childhood experiences with parental love-withdrawal may also shape caregiving and protective responses to offspring or familiar others when confronted with a threat. Although the neural correlates of individual differences in caregiving and protective responses are poorly understood (but see Swain et al., 2014), the presence of a threat may affect the way parents perceive and respond to their child differently based on their own childhood experiences with protective or neglectful attachment figures. Love withdrawal is a parental disciplinary strategy in which the parent’s love and affection is conditional on the child’s behavior and success. Excessive use of love withdrawal is considered psychological maltreatment (Euser et al., 2010) and experiences of love withdrawal have been associated with long-lasting negative outcomes, like fear of failure, low self-esteem, low emotional well-being, and a negative view of parent-child relationships as well as insecure attachment (Bowlby, 1973/1985, p. 243; Assor et al., 2004; Goldstein and Heaven, 2000; Elliot and Thrash, 2004; Renk et al., 2006). Thus, experiencing love-withdrawal has consequences extending beyond the parent-child relationship, affecting ones beliefs about relationships as well as more generalized socio-emotional processes. That personal characteristics and belief systems formed within the parent-child relationship can affect responses to other significant others has convincingly been shown by, e.g., Mikulincer et al. (2005). These authors showed experimentally how feelings of more secure attachment facilitate supporting partners in distress. Previous research has associated childhood experiences of love withdrawal not only with changes in the (neural) processing of and responding to socio-emotional information, including faces (Huffmeijer et al., 2011), but also with changes in effects of external influences, including oxytocin administration, on these processes (Van IJzendoorn et al., 2011; Bakermans-Kranenburg et al., 2012; Huffmeijer et al., 2013).

The present study was a pilot for research to be conducted with mothers, and examined in young-adult females without children of their own whether subliminally presented threatening primes would evoke the expected changes in brain activity in the amygdala and would differentially affect (the neural correlates of) protective responses to pictures of a familiar and an unfamiliar child. In addition, we examined whether these effects would be moderated by empathic concern and self-reported childhood experiences of love-withdrawal. In order to provide a “proof of concept”, we used a homogenous student sample without children. We mimicked maternal reactions by presenting as “own child” the picture of a child face modified to resemble the participant’s face, and combined this with primes depicting neutral and threatening scenes to evoke (the neural correlates of) protective responses. Facial resemblance is a very important cue for kinship (Bressan and Grassi, 2004; Maloney and Dal Martello, 2006) and has been shown to increase “parental” responses such as willingness to invest in a child (e.g., DeBruine, 2004; Platek et al., 2004). Thus, using pictures of children facially resembling the participants (by use of morphing, see “Materials and Methods” Section) is probably the most accurate imitation of an “own” child in participants without children of their own. However, we cannot exclude the possibility that the morphed faces will only be perceived as familiar rather than suggesting biological relatedness.

We focused our analyses on brain regions known to be involved in the processing of threat and face familiarity: the amygdala (involved in threat detection as well as more general salience detection, and responsive to face familiarity in previous studies [Natu and O’Toole, 2011]), inferior frontal gyrus (IFG, implicated in the processing of familiar faces, see for a review Devue and Brédart, 2011; Platek et al., 2008; implicated in affective empathy, Shamay-Tsoory, 2011, and considered part of the mirror neuron system, e.g., Kilner et al., 2009), and superior temporal gyrus (STG, found to be activated in response to unfamiliar compared to personally familiar faces, see Ramon et al., 2015, and involved in Theory of Mind [ToM]). Importantly, these areas have not only been associated with the neural processing of threat and/or familiarity, but the functions mediated by these regions (such as ToM, empathy, affect regulation and mirroring) are also considered critical for parental behavior and involvement (Swain et al., 2014). We expected enhanced amygdala activity in response to threatening primes relative to neutral primes. We expected empathy to moderate this effect, with enhanced amygdala activity in highly empathic individuals. In addition, we hypothesized that IFG activity would be elevated in response to familiar-looking compared to unfamiliar-looking faces, and, conversely, that STG activity would be elevated in reaction to unfamiliar compared to familiar-looking faces. We explored potential moderating effects of experiences of love withdrawal, which might moderate effects of face familiarity or might be associated with the strength of a priming effect on familiar faces in particular. We chose to focus on a limited number of regions of interest (ROIs) to retain sufficient statistical power for testing *a priori* hypotheses, but, as interesting or unexpected effects might occur in other brain regions, we also conducted whole-brain analyses to explore changes in brain activity as a result of the primes, familiarity, empathy, and parental love withdrawal.

## Materials and Methods

### Participants

A total of 49 female undergraduate and graduate students aged 18–28 years (*M* = 21.73, *SD* = 2.55) were invited for two experimental sessions, separated by approximately 4 weeks. The second session was included to study test-retest reliability of fMRI data (to be reported elsewhere); the current study uses data from the first session only. Exclusion criteria were MRI contraindications, pregnancy, current psychiatric and neurological disorders, severe head injury, current alcohol or drug abuse, and chronic use of medication (except contraceptives). Data of four participants were excluded from analysis because of excessive head movements (>3 mm; *n* = 3) or falling asleep during fMRI acquisition (*n* = 1). Our final sample therefore included 45 participants with an average age of 21.82 years (*SD* = 2.61, range: 18–28). The study was approved by the Ethics Committee of the Leiden University Medical Center. All participants signed informed consent at the beginning of the first session and were rewarded with 40€ for participation. None of these participants’ structural MRI scans showed any anomalies.

### Procedure

Participants’ handedness was assessed using van Strien’s (1992) Handedness Questionnaire prior to the first session. Participants were asked to abstain from alcohol and excessive physical activity during the last 24 h and from caffeine during the last 12 h before the start of the session. At the beginning of the session participants completed questionnaires on empathy and parental use of love withdrawal. Subsequently, the MRI procedure was explained and participants were placed in the MRI scanner. Foam inserts were placed between the head coil and the participant’s head to minimize head movements. Within the scanner, participants completed a priming task (see below), during which visual stimuli were projected onto a screen placed outside the opening of the scanner bore. Participants viewed the screen through a mirror fixed to the head coil. At the end of the second session participants were debriefed about the nature of the priming task.

### Questionnaires

#### Handedness Questionnaire

This questionnaire consists of 10 items with regard to hand preference during execution of several tasks (e.g., “Which hand do you use to hold scissors?”) scored on a 3-point scale (left hand, both hands, right hand) ranging from −1 to 1. Total scores can thus vary between −10 and +10. Individuals with a score of +8 or higher are classified as strongly right-handed, whereas individuals scoring −8 or lower are classified as strongly left-handed. Individuals with scores between −8 and +8 are classified as ambidexter (van Strien, 2003). According to this definition, in the current sample, 23 participants were strongly right-handed, 19 were strongly left-handed, and three were ambidexter. We oversampled left-handed participants in order to examine the potential influence of left-handedness on neural activity (to be reported elsewhere).

#### Children’s Report of Parental Behavior Inventory

Participants completed a modified version of the 30-item Children’s Report of Parental Behavior Inventory (CRPBI-30, Schludermann and Schludermann, 1983; Beyers and Goossens, 2003), containing the items of the Acceptance and Psychological Control scales from the original questionnaire and several extra items to measure love withdrawal. The 11-item Love Withdrawal scale consisted of all five items that constitute the Withdrawal of Relations subscale of the 108-item CRPBI (3 of which are also included in the Psychological Control scale of the CRPBI-30; Schludermann and Schludermann, 1983), two items that were adapted from this same questionnaire, and four items that were adapted from the Parental Discipline Questionnaire (PDQ, Hoffman and Saltzstein, 1967; Patrick and Gibbs, 2007). Participants rated how well each item described their mother and father separately (e.g., “My mother was a person who if I’d hurt her feelings, stopped talking to me until I please her again”) on a 5-point Likert scale, ranging from (“not at all”) to (“very well”). We only included the 11-items of the Love Withdrawal subscale in our analyses. Scores for maternal and paternal love withdrawal were summed. After winsorizing the score of one outlier (*z* = 3.61; the new score was computed as the highest score occurring in the rest of the sample plus the difference between the highest and next-highest score, see Tabachnick and Fidell, 2001), the scores were normally distributed with an average score of 18.72 (*SD* = 6.15). Internal consistency of this questionnaire was high (Cronbach’s alpha = 0.91). Adequate validity and reliability of the CRPBI and its subscales were demonstrated (Schludermann and Schludermann, 1983, 1988; Locke and Prinz, 2002) and the Love Withdrawal subscale as used in this study was implemented in earlier research on the consequences of maternal love withdrawal in young adults (Huffmeijer et al., 2011).

#### Interpersonal Reactivity Index

To measure empathy, participants completed the 28-item Interpersonal Reactivity Index, a well validated questionnaire measuring four distinct aspects of empathy (Perspective Taking, Fantasy, Empathic Concern, and Personal Distress; Davis, 1983; De Corte et al., 2007). In the current analyses, we only administered the seven-items of the Empathic Concern subscale, since we were interested in the emotional component of empathy. Participants rated how well each of the items described themselves on a 5-point Likert scale, ranging from 0 (“does not describe me well”) to 4 (“describes me very well”). The data were normally distributed and did not contain any outliers. On average, participants scored 19.36 (*SD* = 3.53) on the Empathic Concern scale. The internal consistency was acceptable (α = 0.67).

Scores on Love Withdrawal and Empathic Concern were not correlated (*r* = 0.00) and could therefore be included as independent predictors in the same analyses.

### Experimental Task

In the scanner, subjects completed a priming task consisting of 234 trials. The priming task was set up in an event-related design. E-prime Software (Psychology Software Tools, 2012) was used for stimulus presentation. All stimuli were shown in the center of the screen on a black background. Forward and backward masking of the primes, using a picture showing a colored, circular pattern, was used on all trials to prevent conscious perception of the primes. The mask matched the dimensions and average luminosity of the primes. During each trial, a fixation cross was presented for 1800–10,600 ms, followed by the mask (presented for 484 ms), a prime (i.e., a neutral or threatening picture) that was presented for 16 ms, and again the mask (presented for 100 ms). Subsequently, an unfamiliar-looking, a familiar-looking or a scrambled face was presented for 2000 ms. Thus, there were six conditions: a familiar-looking face presented after a neutral prime (neutral-familiar), a familiar-looking face presented after a threatening prime (threat-familiar), an unfamiliar-looking face presented after a neutral prime (neutral-unfamiliar), an unfamiliar-looking face presented after a threatening prime (threat-unfamiliar), a scrambled face presented after a neutral prime (neutral-scrambled), and a scrambled face presented after a threatening prime (threat-scrambled). Stimulus sequences (mask-prime-mask-[scrambled] face) were presented in quasi-random order, with the restriction that the same prime could not be presented more than twice in a row, the same face could not be repeated more than four times in a row, and the same condition could not repeat more than twice. In all, 13 neutral and 13 threatening primes were each presented three times with each face, resulting in 39 (3 × 13) trials per condition. To ensure that participants remained alert during the task, they had to press a button in order to continue the task after every 11–13 trials. The average duration of the task was 23 min.

### Primes

The stimuli used as primes were developed by Nummenmaa et al. (2008). To enable comparability between neutral and threatening primes, these authors created pairs of photographs depicting a neutral and a threatening scene, respectively. Each pair was matched on luminosity, global energy, contrast density, and complexity, and showed the same persons in comparable proximity to each other. Each photograph portrayed two persons. On threatening photographs, interpersonal attack scenes (e.g., one person strangling the other) were shown, whereas non-emotional situations (e.g., two persons having a conversation) were depicted on neutral photographs.

We selected 13 pairs out of the 37 pairs of threatening and neutral pictures (Nummenmaa et al., 2008): an independent sample of 15 participants were presented with the pictures for 16 ms, with forward and backward masking as described above, and asked to press one button if they were sure a neutral picture had been presented, a second button if they were sure a threatening picture had been presented (they were instructed to press these buttons only if they had seen the picture and were sure of its contents), and a third button if they had not seen the picture or were unsure of its contents. This was done to test whether the neutral and threatening pictures were visible for the participants when these pictures were presented for 16 ms. Ideally, the participants should not be able to consciously perceive and identify the pictures, since our goal was to investigate subliminal processing of neutral and threatening stimuli. Therefore, only pictures that were not identified as neutral or threatening above chance levels (i.e., pictures for which significantly more than 50% of participants answered “unsure”) were selected for use in the current study. Another independent sample of 28 participants was used to rate the 13 pairs of pictures for valence and arousal. Threatening photographs (*M* = 8.40, *SD* = 0.22) were rated as significantly more negative than neutral photographs (*M* = 4.48, *SD* = 0.60; *t*_(12)_ = −23.90, *p* < 0.01, *d* = −8.67), on a scale ranging from 1 (“positive”) to 9 (“negative”). Moreover, on a scale ranging from 1 (“affected”) to 9 (“calm”), threatening primes (*M* = 3.43, *SD* = 0.41) evoked significantly more arousal than neutral primes (*M* = 7.31, *SD* = 0.33; *t*_(12)_ = 21.62, *p* < 0.01, *d* = 10.43).

At the end of the second session, participants in the current study were asked whether they had seen any of the pictures presented in between the masks (i.e., the primes). Twenty-six participants (58%) indicated that they had noticed the pictures. Subsequently, these participants were asked to indicate which of several items (e.g., “truck”, “adults”) they had seen in the pictures. Some of these items had actually been present in the pictures, others had not. None of the participants performed above chance level, the participants selected seen and unseen items with equal probability.

### Facial Stimuli

Pictures of unfamiliar- and familiar-looking children were created by morphing the photograph of a child’s face (unfamiliar to the participant) with: (i) a photograph of an unknown female’s face and (ii) a photograph of the participant’s own face. Prior to the first session, participants were asked to provide a full-color digital photograph of themselves that met the following criteria: picture on a light and uniform background, showing their face (full frontal) and neck only, with a neutral facial expression, and no piercings, make-up or glasses. Full color, full frontal photographs of two female faces (both Caucasian and unfamiliar to the participant, aged 24 and 25 year, neutral facial expression, no jewelry or glasses) were used to create the unfamiliar-looking morphs. For half of the participants, female face 1 was used to create the unfamiliar-looking morph for session one and female face 2 was used to create the unfamiliar-looking morph for session two, and for the other half vice versa. Full color, full frontal photographs of six 9–11 year old children (three boys and three girls, all Caucasian [but slightly varying in skin color], all unfamiliar to the participants, with neutral facial expression, no jewelry or glasses) were available for morphing. For half the participants (*n* = 21 for the current sample) morphs were created with the picture of a female child and for the other half (*n* = 24 for the current sample) morphs were created with the picture of a male child. Within genders, the child that best matched the participant’s skin color and face-shape was selected for ease of morphing. Both unfamiliar-looking and familiar-looking morphs were created with the photograph of the same child. One familiar-looking and two-unfamiliar-looking morphs were created for the two sessions. We did not use the same unfamiliar-morph for both sessions, since this would have led to increased familiarity with the unfamiliar-looking face in session two compared to session one.

Prior to morphing, all photographs were resized to 448 × 560 pixels and edited using Adobe Photoshop CS: External features (i.e., hair and ears) were removed and the pictures were framed on a black background. Morphing was then performed using Fantamorph 5 Deluxe, such that the picture of the familiar-looking child consisted for 50% of the participant’s face and for 50% of an unknown child’s face, and the picture of the unfamiliar-looking child consisted for 50% of the unknown female’s face and for 50% of the child’s face. The resulting pictures appear to present children slightly older than the 9–11 year olds used for morphing. An independent sample of 15 participants rated the age of the unfamiliar-looking morphs as 13.80 years (*SD* = 1.66) and the familiar-looking morphs as 14.40 years (*SD* = 1.60) on average (*p* > 0.05). Finally, a scrambled face was created for each participant from the familiar-looking morph by randomly rearranging blocks of 9 × 9 pixels using Matlab R2012B.

At the end of the second session, participants in the current study evaluated how much the familiar-looking and unfamiliar-looking faces used during the priming task resembled themselves on a scale ranging from 0% resemblance to 100% resemblance. On average, the participants reported a similarity of 38.07% (*SD* = 13.38%) with the familiar and 6.40% (*SD* = 6.84%) with the unfamiliar morphs. The difference in perceived similarity was significant with a large effect size (*t*_(44)_ = 15.82, *p* < 0.01, *d* = 2.98).

### Image Acquisition

Images were acquired at the Leiden University Medical Center on a 3-T Philips Achieva MRI system (Philips Medical Systems, Best, Netherlands) with a 32-channel SENSE (Sensitivity Encoding) head coil. An event-related design with 680 T2*-weighted whole-brain echo planar images (EPI, repetition time (TR) = 2200 ms, echo time (TE) = 30 ms., flip angle = 80°, 38 transverse slices, descending acquisition order, voxelsize = 2.75 × 2.75 × 3.025 mm^3^ with a 10% interslice gap, field of view (FOV) = 220 × 114.675 × 220 mm^3^) was used for the functional scans. To avoid magnetic saturation effects, the first four functional scans were discarded. In addition, an anatomical 3D T1-weighted scan (TR = 9.825 ms, TE = 4.605 ms, flip angle = 8°, 140 transverse slices, voxelsize 0.875 × 0.875 × 1.2 mm^3^, FOV = 224 × 168 × 177.333 mm^3^) and a high-resolution T2*-weighted EPI-image (TR = 2200 ms, TE = 30 ms, flip angle = 80°, 84 transverse slices, voxel size = 1.964 × 1.964 × 2 mm^3^, FOV = 220 × 168 × 220 mm^3^) were obtained for coregistration purposes.

### fMRI Data Analysis

Data-analyses were performed using FSL (FMRIB’s Software Library^1^) FEAT (FMRI Expert Analysis Tool) version 5.0.4, part of Jenkinson et al. (2012) and Smith et al. (2004). The following pre-statistics processing steps were carried out: motion correction using MCFLIRT (Jenkinson et al., 2002), non-brain removal (BET; Smith, 2002), spatial smoothing using a Gaussian kernel with a full-width-at-half-maximum of 6 mm, and high-pass temporal filtering with a high-pass filter cutoff of 100 s.

Functional images were registered to the high-resolution EPI-image, which was then registered to the 3D T1-weighted scan, and then to the 2 mm isotropic MNI-152 standard space image (T1 standard brain averaged over 152 subjects; Montreal Neurological Institute, Montreal, QC, Canada; Jenkinson et al., 2002). Functional activity in response to the stimuli was investigated using general linear model analysis in native space. Because primes and masks were presented for very short durations and time-locked to the presentation of the faces, hemodynamic responses to the individual stimuli within a mask-prime-mask-face sequence overlap extensively and sum to a total, summed hemodynamic response to the stimulus sequence. Assuming that responses to the masks in a given brain area do not vary systematically across the different conditions (as these are defined by different types of primes and the faces, but the masks are always the same), this summed response may vary depending on the response to the primes and faces. We thus treated the presentation of mask-prime-mask-face as a single stimulation period, and thus the different conditions (threat-familiar, threat-unfamiliar, threat-scrambled, neutral-familiar, neutral-unfamiliar, and neutral-scrambled) and the participants’ responses were modeled as seven explanatory variables using the Custom (3 column format) wave function and convolved with a double gamma hemodynamic response function. The temporal derivatives of the explanatory variables were included in the model, yielding 14 regressors. Subsequently, individual lower-level contrast images (see below) were submitted to higher-level mixed effects (FLAME 1 + 2) group ROI and whole-brain analyses. Group means for ROIs and whole-brain analyses were tested using F-tests. All statistical images were thresholded using clusters determined by *Z* > 2.3 (*F*-values are automatically converted to *z*-statistics) and a cluster-corrected significance threshold of *p* < 0.05 (Worsley, 2001)^2^.

Before evaluating our main hypotheses, a preliminary analysis was conducted to check whether faces activated known face processing areas such as the fusiform gyrus more than scrambled stimuli. For this purpose, the contrast face (i.e., neutral-familiar, neutral-unfamiliar, threat-familiar, threat-unfamiliar) > scrambled face was tested. In the preliminary analysis, no confound regressors or continuous predictors were added to the model and only whole-brain analysis was conducted. Results of the preliminary analysis, showing that the facial stimuli reliably activated face processing areas as expected, can be found in the Supplementary Materials.

To evaluate our main hypotheses, separate whole-brain and ROI-analyses were performed to test for: (i) differences in brain activity in response to stimulus sequences in which faces were presented with a neutral prime and sequences in which faces were presented with a threatening prime; (ii) differences in brain activity in response to familiar and unfamiliar faces; and (iii) interactions between the type of face and the type of prime. For these analyses, five contrasts of interest were calculated: (1) familiar (threat-familiar and neutral-familiar) vs. unfamiliar (threat-unfamiliar and neutral-unfamiliar); (2) threatening (threat-familiar and threat-unfamiliar) vs. neutral (neutral-familiar and neutral-unfamiliar); (3) (threat-familiar vs. neutral-familiar) vs. (threat-unfamiliar vs. neutral-unfamiliar); (4) threat-familiar vs. neutral-familiar; and (5) threat-unfamiliar vs. neutral-unfamiliar. The first contrast tested for differences in brain activity in response to viewing familiar-looking faces compared to unfamiliar-looking faces. Because the type of face presented may be expected to affect only the hemodynamic response to the face stimulus (as the prime is presented before it), the areas identified respond differently to familiar and unfamiliar faces. The second contrast tested for effects of the primes, i.e., differences in brain activity in response to presentation of sequences including neutral primes compared to sequences including threatening primes. Because the type of prime presented could theoretically affect the hemodynamic response to both the prime itself and the face stimulus, this contrast will identify both brain regions that respond differentially to the neutral and threatening primes (i.e., areas involved in processing the primes) and brain areas that respond differently to faces (regardless of whether this was a familiar or unfamiliar face) depending on the type of prime (i.e., a priming effect on face processing). In case of significant effects, comparisons to sequences including a scrambled stimulus instead of a face are used to distinguish between these two options. The third contrast tested for the interaction (i.e., variation in the effect of familiarity depending on the type of prime and/or variation in the effect of priming depending on face familiarity), and significant results for contrasts 4 and 5 were only interpreted in areas where contrast 3 was significant. F-tests were used to evaluate the hypotheses of the whole-brain and ROI-analyses. Scores on love withdrawal and empathic concern were included as continuous predictors and handedness was added to the model as a confound regressor.

The ROI analyses were performed on bilateral amygdala, bilateral inferior frontal gyrus (IFG) and bilateral STG to test our *a priori* hypotheses. Three higher-level analyses, restricted to bilateral amygdala, IFG and STG respectively, were conducted to investigate activity in these regions with maximized statistical power by limiting the number of statistical tests to the investigated ROI. The Harvard-Oxford Subcortical Structures Atlas was used to define the ROI for the amygdala and the Harvard-Oxford Cortical Structures Atlas (both implemented in FSL version 5.0.4) was used to define ROIs for the IFG and the STG. Three masks were created in 2 mm isotropic MNI-152 standard space (Jenkinson et al., 2002), consisting of voxels belonging to the left or right amygdala, IFG and STG respectively with a probability of at least 25%. Exploratory whole-brain analyses were performed to investigate brain activity in regions other than the *a priori* ROIs.

As use of caffeine may have an influence on brain activity as measured with fMRI (Liu et al., 2004; Liau et al., 2008; Perthen et al., 2008; Chen and Parrish, 2009), we reran the ROI and whole brain analyses testing effects of face and prime type excluding participants (*n* = 7) who did not comply with the request to abstain from caffeine during the last 12 h before the study. Using this sensitivity analysis, we evaluated whether effects in the total sample were replicated in the sample without caffeine-using respondents.

## Results

All significant clusters were defined by *Z* > 2.3 and a cluster-corrected significance threshold of *p* < 0.05 (Worsley, 2001).

### ROI Analyses

**Figure 1:**
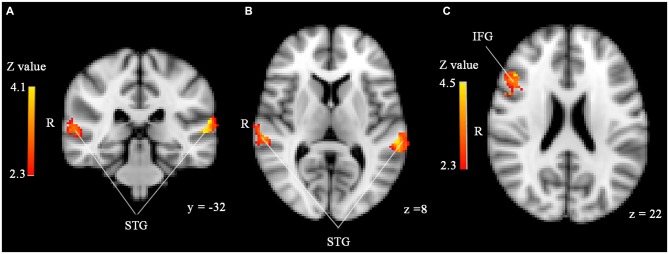
**(A,B)** Significantly enhanced activity in bilateral superior temporal gyrus (STG) in response to unfamiliar compared to familiar faces in the sample with 45 participants. **(C)** Significantly enhanced activity in right inferior frontal gyrus (IFG) in response to familiar compared to unfamiliar faces. ROI analyses, *p* < 0.05, corrected by cluster threshold (*Z* > 2.3).

Significant effects of face familiarity were found in both the IFG and STG (see Figure 1): familiar-looking faces elicited greater brain activity than unfamiliar-looking faces in the right IFG (size = 220, *Z-max* = 4.54, MNI coordinates *x, y, z* (mm) = 46, 26, 22), whereas unfamiliar-looking faces elicited greater brain activity than familiar-looking faces in bilateral STG (cluster 1 [left]: size = 304, *Z-max* = 4.1, MNI coordinates *x, y, z* (mm) = −62, −32, 14, cluster 2 [right]: size = 182, *Z-max* = 3.53, MNI coordinates *x, y, z* (mm) = 64, −26, 10). No effects of familiarity were found in the amygdala, and we did not find significant activity differences between stimuli preceded by threatening and neutral primes or any familiarity*prime type interaction in any of the ROIs. Love withdrawal and empathic concern did not affect brain activity in any of the ROIs either.

### Whole-Brain Analyses

**Figure 2:**
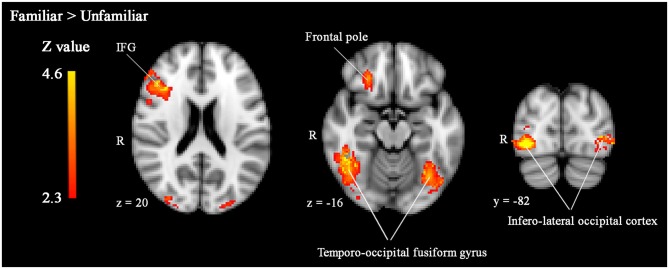
**Significantly enhanced activity in right IFG, bilateral temporo-occipital fusiform gyrus, and infero-lateral occipital cortex in response to familiar compared to unfamiliar faces.** Whole-brain analyses, *p* < 0.05, corrected by cluster threshold (*Z* > 2.3).

To explore effects of threat priming and familiarity in regions outside our regions of interest, we performed whole-brain analyses. Results of these analyses revealed more widespread effects of face familiarity on brain activity (see Table 1). As illustrated in Figure 2, familiar-looking faces elicited greater brain activity in a cluster including not only the right IFG, but also parts of the right middle frontal gyrus (MFG), frontal pole, and insular cortex (cluster 2: size = 1559, peak *Z-max* = 4.54, MNI coordinates *x, y, z* (mm) = 46, 26, 22; see Figure 2). We also found increased activity in response to familiar- compared to unfamiliar-looking faces in bilateral clusters including the occipital pole, infero-lateral occipital cortex, and the temporo-occipital fusiform gyrus (cluster 3 [right]: size = 1627, peak *Z-max* = 4.63, MNI coordinates *x, y, z* (mm) = 40, −72, −10, cluster 1 [left]: size = 1182, peak *Z-max* = 4.29, MNI coordinates *x, y, z* (mm) = −30, −90, 6).

**Table 1:** **MNI Coordinates and *Z*-max values for regions with significant main effects for face familiarity and for regions in which love withdrawal and empathic concern interact with effects of face familiarity**.

Experimental effect	Cluster number	Size (# voxels)	Region	*Z-max*	MNI coordinates for for *Z*-max		
					*x*	*y*	*z*
Familiar > unfamiliar	3	1627	Right infero-lateral occipital cortex	4.63	40	−72	−10
	2	1559	Right MFG	4.54	46	26	22
	1	1182	Left infero-lateral occipital cortex	4.29	−30	−90	6
Unfamiliar > familiar	4	2815	Left planum temporale	4.57	−56	−30	10
	3	1794	Right postcentral gyrus	3.86	24	−34	60
	2	1504	Right planum temporale	4.09	48	−30	16
	1	765	Cuneus	3.85	0	−78	26
Familiar > unfamiliar^LW+^	1	1008	Right infero-lateral occipital cortex	4.22	34	−80	0
Familiar > unfamiliar^EC+^	1	593	Left frontal pole	3.68	−22	56	36

*Note: Familiar > unfamiliar^LW+^, Positive correlation between love-withdrawal and the activity difference in response to familiar and unfamiliar (i.e., familiar > unfamiliar) faces. Familiar > unfamiliar^EC+^, Positive correlation between empathic concern and the activity difference in response to familiar and unfamiliar (i.e., familiar > unfamiliar) faces*.

**Figure 3:**
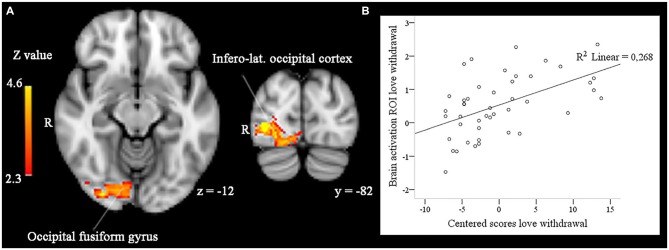
**(A)** In right infero-lateral occipital cortex and right occipital fusiform gyrus the effect of face familiarity (enhanced activity to familiar compared to unfamiliar faces) is significantly and positively related to participants’ scores on love withdrawal in whole-brain analyses, *p* < 0.05, corrected by cluster threshold (*Z* > 2.3). **(B)** Scatterplot between the activity difference (familiar > unfamiliar) found in these areas and participants’ centered scores on love withdrawal.

In addition, love withdrawal interacted with the effect of familiarity in a partially overlapping cluster including the right infero-lateral occipital cortex, occipital fusiform gyrus, and occipital pole (size = 1008, peak *Z-max* = 4.22, MNI coordinates *x, y, z* (mm) = 34, −80, 0). As illustrated in Figure 3, the effect of familiarity was larger (i.e., a larger difference in brain activity in response to familiar-looking compared to unfamiliar-looking faces) for participants reporting more love withdrawal.

**Figure 4:**
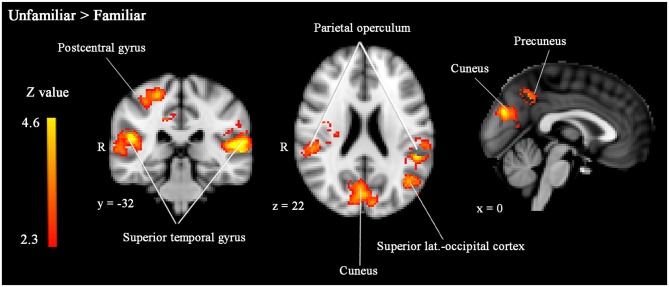
**Significantly enhanced activity in bilateral STG, right postcentral gyrus, bilateral parietal operculum, left superior lateral occipital cortex, bilateral cuneus and left precuneus in response to unfamiliar compared to familiar faces.** Whole-brain analyses, *p* < 0.05, corrected by cluster threshold (*Z* > 2.3).

Unfamiliar-looking faces compared to familiar-looking faces evoked increased activity bilaterally in clusters including not only the STG, but also the posterior division of the supramarginal gyrus, and the parietal operculum, and extending anteriorly into the planum temporale (cluster 4 [left]: size = 2815, peak *Z-max* = 4.57, MNI coordinates *x, y, z* (mm) = −56, −30, 10; cluster 2 [right]: size = 1504, peak *Z-max* = 4.09, MNI coordinates *x, y, z* (mm) = 48, −30, 16). In addition, unfamiliar-looking faces compared to familiar-looking faces elicited heightened activity in a cluster including the right postcentral gyrus, right superior parietal lobe, and bilateral precuneus (cluster 3: size = 1794, peak *Z-max* = 3.86, MNI coordinates *x, y, z* (mm) = 24, −34, 60) and in bilateral cuneus (cluster 1: size = 765, peak *Z-max* = 3.85, MNI coordinates *x, y, z* (mm) = 0, −78, 26). These clusters are presented in Figure 4.

Empathic concern interacted with face familiarity in a frontal area (frontal pole: size = 593, peak *Z-max* = 3.68, MNI coordinates *x, y, z* (mm) = −22, 56, 36) far at the outside of the brain and not overlapping with any of the significant clusters described above, suggesting artifactual activity.

Similar to the ROI analyses, the whole-brain analyses did not reveal any significant activity differences between stimuli preceded by threatening and neutral primes or any familiarity*prime type interaction.

### Analyses with 38 Participants

The clusters that we found in the ROI and whole-brain analyses in the total sample of 45 participants were largely replicated with the 38 participants who abstained from caffeine use. In the smaller sample, however, familiar-looking compared to unfamiliar-looking faces evoked enhanced activity only in right occipital pole, infero-lateral occipital cortex, and temporo-occipital fusiform gyrus and not bilaterally as in the sample with 45 participants. This small difference may be due to lower statistical power in the smaller sample.

## Discussion

In this study, we investigated the processing of subliminally presented threatening primes and their effects on neural responses to pictures of a familiar (and potentially “own”) and unfamiliar child in a homogenous student sample. In addition, we studied moderating effects of empathy and experiences of love-withdrawal. Since we were particularly interested in parental protective reactions in the presence a potential threat, we combined pictures of faces of familiar-looking children with primes that depicted threatening scenes. Contrary to our expectations, we did not find evidence of a priming effect, nor of any interaction between empathy or experienced love withdrawal and priming. The primes used in our study depicted fairly complex neutral and threatening scenes, showing multiple people and objects. It is possible that these images were too complex for the brain to extract the threatening or neutral content fast and efficiently, and that preconscious processing of the threat thus did not occur. Earlier studies on subliminal priming usually used less complex stimuli (e.g., fearful or angry facial expressions; e.g., Morris et al., 1998; Whalen et al., 1998; Almeida et al., 2013). However, if the brain possesses a specialized threat-detection system to enable fast and automatic responses to environmental threats, we would expect such a system to be able to process threatening stimuli with various contents and complexities.

An absence of priming effects on face processing may not only be due to the complexity of the scenes used as primes. It is also possible that the scenes did not induce protective reactions. It seems natural, however, that parents or adults in general look after children when they witness or expect threatening events and their own survival is not immediately at stake. Although caregiving responses may be weaker when the child is not in distress (i.e., crying) or when the threat is aimed at the adult, the hypothesis that neural differences can be observed if the subliminal threat is really processed may still be warranted. Interestingly, results of some recent studies actually do not provide much support for the existence of a human brain system capable of preconscious processing of threatening information (see Pessoa and Adolphs, 2010; Hoffmann et al., 2012). It should be noted that in earlier studies of “subliminal” processing of affective information primes were often presented for 30 ms or longer (Morris et al., 1998; Whalen et al., 1998; Dimberg et al., 2000; Li et al., 2008). Participants differ in their sensitivity to threatening stimuli, but reliable detection of fearful faces has been observed with presentation durations of only 17 ms (Pessoa et al., 2005). This suggests that priming may not have been completely subliminal in the previous experiments with prime presentations of approximately 30 ms. Obviously, what is needed is replication of our study with subliminal stimuli of varying duration and involving a more direct threat to the child or, alternatively, with supraliminal threat stimuli.

We also investigated the effects of face familiarity on neural activity. As hypothesized, we found enhanced activity in response to familiar-looking faces in the IFG, extending into the MFG and insular cortex. Enhanced activity in IFG and MFG is frequently seen in familiar face processing (Gobbini and Haxby, 2006; Platek and Kemp, 2009; Taylor et al., 2009). In addition, enhanced activity of these brain areas is frequently found in response to pictures or videos of an own vs. other/unfamiliar child (Bartels and Zeki, 2004; Noriuchi et al., 2008; Kuo et al., 2012; Wittfoth-Schardt et al., 2012). These effects may be associated with the role of these brain areas in self-referential processing: when confronted with (the face of) someone who physically resembles the self (whether due to kinship or otherwise) concepts relating to the self are automatically activated. This human tendency to extrapolate from physical, “outer”, resemblance to psychological, “inner”, resemblance plays an important role in the understanding (including empathic understanding) of others (see for a review Devue and Brédart, 2011) Importantly, these as well as other processes in which the IFG and insula have an important role (e.g., emotion-regulation) are very important for parental behavior. In fact, Swain et al. (2014) have given these areas an important role in their model of the “parental brain”. We also found enhanced activity in occipital and temporal (i.e., occipital pole, infero-lateral occipital cortex, and fusiform gyrus) areas involved in visual, and, more specifically, face processing (Haxby et al., 2000; Natu and O’Toole, 2011). Our findings suggest preferential processing of the familiar-looking faces. Both of these effects fit well with known processing advantages of stimuli associated with own compared to unfamiliar children in parents (Leibenluft et al., 2004).

Love withdrawal moderated the effect of familiarity in right hemisphere face processing areas (infero-lateral occipital cortex and occipital fusiform gyrus). Participants reporting more love withdrawal showed larger differences in brain activity in response to familiar-looking vs. unfamiliar-looking faces. Interestingly, changes in the neural processing of facial stimuli in young adults reporting high maternal love withdrawal have been observed before (e.g., Huffmeijer et al., 2011). Experiences of love withdrawal create a mental link between behavior and relational consequences, and they compromise the security of the parent-child attachment relationship, which becomes conditional on the child’s behavior (Goldstein and Heaven, 2000; Assor et al., 2004; Elliot and Thrash, 2004; Renk et al., 2006). We suggest that the increased salience and relevance of the parent-child relationship may generalize to relationships more generally and increase the processing of information relevant to those relationships, in particular relationships with other family members, including own children. The enhanced brain activity seen in participants with high scores on love withdrawal to familiar-looking faces, designed to appeal to a kinship bond, may be a neural signature of this processing enhancement.

In contrast to familiar-looking faces, unfamiliar-looking faces enhanced activity in bilateral STG, and in whole brain analyses this activity extended anteriorly from the planum temporale into the parietal operculum and the posterior part of the supramarginal gyrus. In addition, unfamiliar-looking faces elicited enhanced activity in the right postcentral gyrus, right superior parietal lobe, and bilateral cuneus and precuneus. These regions are part of the brain’s socio-emotional networks and they are, in particular the superior temporal sulcus (STS), involved in ToM processes. ToM refers to the cognitive capacity to attribute mental states (e.g., desires, intentions) to others and to predict others’ behaviors from these mental states (Frith and Frith, 1999; Schurz et al., 2014). Although contrasting results exist in the literature (Leibenluft et al., 2004), several previous studies have observed decreased activity in the STS in response to familiar faces (Ramon et al., 2015) and to pictures of mothers’ own children (Bartels and Zeki, 2004) compared to unfamiliar faces. Decreased activity in brain areas supporting ToM in response to familiar compared to unfamiliar faces may be explained by reduced effort, i.e., due to for example self-referential processing (see above) it is easier to estimate the mental state of someone familiar or similar to the self and by a lower need to investigate the social validity, i.e., a reduced need to thoroughly assess/estimate the mental state or intentions of familiar persons, as suggested by Bartels and Zeki (2004).

Future research should also take some limitations of the current study into account. The most important limitation is of course the use of morphed faces instead of faces of own offspring. The difference between the “own” and unfamiliar children’s faces was physical resemblance (looking familiar). Although the participants reported afterwards that the familiar faces were much more similar to their own faces than the unfamiliar faces and physical resemblance is a kinship cue, replication with faces of real offspring is needed to disentangle effects of biological relatedness and familiarity or physical resemblance in the absence of a kinship bond. Second, the current design did not allow for separate modeling of hemodynamic responses to primes and faces. Because primes and masks were presented for very short durations and time-locked to the presentation of the faces, hemodynamic responses to the individual stimuli within a mask-prime-mask-face sequence overlapped extensively, requiring the modeling of a single, summed hemodynamic response to each stimulus sequence. Although all relevant processes (processing of the primes, processing of the faces, and effects of priming on face processing) could be separated by comparing responses to the different stimulus sequence conditions, it is worthwhile to consider the inclusion of conditions in which either the prime or the face is omitted from the stimulus sequence. Although this would lengthen the paradigm, such a design would allow for separate modeling of responses to primes and faces which may lead to a less complicated analysis approach. Third, we used self-report questionnaires to investigate parental love withdrawal and empathy. There are obvious limitations to the accuracy and reliability of participants’ self-reports. Furthermore, we chose to focus our analyses on the affective component of empathy captured by the Empathic Concern scale of the Interpersonal Reactivity Index (IRI). Future studies may also focus on other empathy dimensions, such as the cognitive component or the tendency to experience personal distress. In addition, the participants in our sample reported relatively low levels of experiences of love withdrawal and were generally psychologically healthy. It may be interesting to replicate the study within clinical samples, e.g., in parents reporting experiences of (emotional) abuse or those with post-traumatic stress symptoms. These experiences and symptoms have been related to hyper-vigilance and arousal (van Harmelen et al., 2013; Stark et al., 2015), and individuals with post-traumatic symptoms in particular seem to have enhanced amygdala responses to threat (Stark et al., 2015). Thus, they may be more sensitive to (supraliminally or subliminally presented) threat primes, which may lead to altered priming effects. Third, as this was a pilot study focusing on women without children, for which pictures of an “own” child were artificially created, future studies should certainly focus on parents’ neural responses to their own and unfamiliar children in the presence and absence of threat. As we found no evidence of subliminal priming effects, replication studies might want to modify the priming design. Less complex stimuli (e.g., angry or fearful vs. neutral faces) could be used, as the neutral and threatening primes used in our study were perhaps too complex. Primes could also be presented supraliminally. In fact, ideally the primes should be both sub- and supraliminally, to directly compare brain activity seen in both conditions and to shed light on the possibility of subliminal threat processing. Finally, a behavioral measure (e.g., reaction time) of the priming effect could be included to directly compare changes in brain activity to changes in behavior.

So far, our results question the effectiveness of subliminal threat-priming. As others have questioned the existence of a fast and automatic threat processing system, too (see Pessoa and Adolphs, 2010; Hoffmann et al., 2012), we feel that this is an important issue that deserves attention in future research. In addition, our results again illustrate the profound impact that experienced parenting strategies such as love withdrawal may have, even at the level of neural information processing. Although changes in neural activity and the preferential processing of familiar vs. unfamiliar faces are not inherently adaptive and desirable or maladaptive and undesirable (as this ultimately depends on the characteristics and demands of the situation or context in which they occur), parental use of love withdrawal may generally be considered undesirable because of its behavioral consequences (see e.g., Assor et al., 2004; Renk et al., 2006). It certainly has to be considered insensitive parental behavior that elevates the chance for an insecure attachment relationship to develop (Bowlby, 1973/1985). The fact that even relatively “mild” negative parenting experiences, such as the levels of love withdrawal reported by our participants, are associated with changes in very basic neural processes only adds to the importance of the early parenting environment and attachment relationship for individual development.

## Author Contributions

All authors listed, have made substantial, direct and intellectual contribution to the work, and approved it for publication.

## Conflict of Interest Statement

The authors declare that the research was conducted in the absence of any commercial or financial relationships that could be construed as a potential conflict of interest.

## Footnotes

^1^www.fmrib.ox.ac.uk/fsl

^2^Due to the large number of voxels analyzed, multiple testing is a well-known problem inherent to fMRI research. We chose to use cluster-extent based correction to correct for multiple testing. This correction procedure combines a threshold for results at individual voxels (i.e., for a cluster of voxels to be considered significantly activated all *z*-values at individual voxels within that cluster must exceed a certain value, in this case 2.3; *t*- and *F-values* are automatically converted to *z*-values) with requirements for the size of the cluster (i.e., a cluster is only considered significant if it consists of a sufficient number of voxels), in such a way that the probability of finding active clusters under the null hypothesis is smaller than 0.05 (i.e., α). To offer some indication of the robustness of statistical findings it is common to report the *z*- and *p*-values (α) employed in the cluster-based correction procedure, as well as the cluster size (in number of voxels) of significant clusters and the maximum *z*-value (i.e., *Z*-max) found among individual voxels in each significant cluster.

## Abbreviations

fMRI: Functional magnetic resonance imaging
ROI: Region of interest
IFG: Inferior frontal gyrus
MFG: Middle frontal gyrus
STG: Superior temporal gyrus
ToM: Theory of mind.
