# Supplementary material for: Neural Processing of Familiar and Unfamiliar Children’s Faces: Effects of Experienced Love Withdrawal, but No Effects of Neutral and Threatening Priming
Source: Front Hum Neurosci. 2016 May 26;10:231. doi: 10.3389/fnhum.2016.00231 (PMC4881397; doi:10.3389/fnhum.2016.00231)
Supplement: Supplementary file 1 [file Table_1.pdf]

Supplementary Material

Neural Processing of Familiar and Unfamiliar Children's Faces: Effects of Experienced Love Withdrawal, but no Effects of Neutral and Threatening Priming

Esther Heckendorf[1, 2], Renske Huffmeijer*[1, 2], Marian J. Bakermans-Kranenburg[1, 2] and Marinus H. Van IJzendoorn[1, 2]

*** Correspondence: Dr. Renske Huffmeijer: rhuffmeijer@fsw.leidenuniv.nl**

1 Supplementary Data

1.1 Preliminary analyses

To confirm that our facial stimuli reliably activated face processing areas, we contrasted brain activity in response to familiar-looking and unfamiliar-looking faces with brain activity in response to scrambled faces. Group means were tested using one-sample t-tests. All statistical images were thresholded using clusters determined by $Z > 2.3$ (t-values are automatically converted to z-statistics) and a cluster-corrected significance threshold of $p < 0.05$ (Worsley, 2001). As shown in Table 2, faces elicited heightened brain activity in bilateral occipital and temporal areas known to be particularly involved in face processing, including the infero-lateral occipital cortex and occipito-temporal fusiform gyrus (clusters 3 and 4). In addition, faces elicited heightened activity in bilateral intracalcarine and supracalcarine cortex (cluster 2), and in several right hemisphere areas that are part of the brain's socio-emotional networks, including a cluster encompassing parts of the MFG, IFG, insular cortex, and precentral gyrus (cluster 6), as well as in a right occipito-parietal cluster (cluster 5, including the superior lateral occipital cortex, and angular gyrus, extending into superior parietal areas), and in a cluster including parts of the right orbitofrontal cortex, amygdala, putamen, and brain stem (cluster 1).

2 Supplementary Figures and Tables

2.1 Supplementary tables

Table 2. MNI coordinates and Z-max values for clusters significantly activated in response to unfamiliar- and familiar-looking faces compared to scrambled stimuli (contrast: face > scrambled).

Cluster	Size	Region	*Z-max*	MNI coordinates for Z-max		
				x	y	z

6	4369	Right MFG and IFG	5.12	48	30	20
5	2746	Right superior parietal lobe	5.25	36	-52	46
4	2576	Right temporal occipital fusiform	5.64	44	-52	-22
3	1381	Left infero-lateral occipital cortex	6.29	36	-82	-2
2	1188	Bilateral intracalcarine cortex	5.06	-14	-64	6
1	1073	Right amygdala	4.18	14	-6	-16
